# Telerehabilitation for visual field defects with a multisensory training: a feasibility study

**DOI:** 10.1186/s12984-025-01573-4

**Published:** 2025-02-24

**Authors:** Nadia Bolognini, Lorenzo Diana, Angela Rossetti, Lisa Melzi, Gianpaolo Basso, Vittorio Manzo, Francy Cruz-Sanabria, Gabriella Cammarata, Franco Cernigliaro, Stefania Bianchi Marzoli, Francesca Tinelli, Simona Fiori, Carlotta Casati

**Affiliations:** 1 Department of Psychology, University of Milano-Bicocca and NeuroMI, Piazza Ateneo Nuoco 1, Milan, 20126 Italy; 2 Laboratory of Neuropsychology, Department of Neurorehabilitation Sciences, IRCCS Istituto Auxologico Italiano, Milan, Italy; 3 Neuro-Ophthalmology Center and Ocular Electrophysiology Laboratory, IRCCS Istituto Auxologico Italiano, Milan, Italy; 4 School of Medicine and Surgery, University of Milano-Bicocca, Monza, Italy; 5 Fondazione IRCCS San Gerardo Dei Tintori, Monza, Italy; 6 Department of Radiology, IRCCS Istituto Auxologico Italiano, Milan, Italy; 7 Department of Developmental Neuroscience, IRCCS Fondazione Stella Maris, Pisa, Italy; 8 Neuroscience Department, Meyer Children’s Hospital, Florence, Italy

**Keywords:** Telerehabilitation, Visual field defects, Hemianopia, Audio-visual training, Oculomotor compensation, Visual scanning

## Abstract

**Background:**

Acquired homonymous visual field defects (HVFDs) result in significant disability, reducing quality of life. Spontaneous recovery occurs within the first months, then the likelihood of vision recovery decreases, making rehabilitation necessary. HVFDs rehabilitation is typically lengthy and intensive, done on an outpatient basis, hardly compatible with the return to everyday life. Telerehabilitation represents an option for continuing the therapy in the chronic phase of the disease, offering long-term support after hospital discharge. It also allows individuals with HVFDs to exercise independently, intensively, and actively at home, in a familiar environment, under remote supervision. However, the efficacy of telerehabilitation for chronic HVFDs in adults still requires empirical support.

**Methods:**

This single-arm clinical trial assesses the efficacy of a home-based, remote-supervised, compensatory audio-visual training (AVT) in 26 adults with chronic HVFDs following a brain lesion. Immediate and long-term (up to 6 months) effects on visual field scanning, reading, activities of daily living and mood were assessed. Predictors of treatment-induced gains were also investigated considering behavioral, neuro-ophthalmological (visual field perimetry and visual evoked potentials) and neuroradiological variables (structural imaging of grey- and white-matter damages). Finally, the efficacy of the home-based AVT was compared to that of its in-person version (16 new participants with chronic HVFDs).

**Results:**

Home-based AVT improves accuracy and speed of visual search, reading, mood, and disability in the activities of daily living, with improvements persisting up to 6 months after the end of the training (baseline vs. post-training assessments, all ps < 0.04). Post-treatment gains correlate with the severity of visual search deficit and the efficiency of multisensory integration (rs = -0.7/-0.5, all ps < 0.04). Neuro-ophthalmological and neuroradiological (structural connectivity) parameters are unaffected by the AVT, in line with its compensatory nature, although being associated to its efficacy (all ps < 0.03). Finally, the telerehabilitation version of the AVT produces effects comparable to the in-person AVT.

**Conclusion:**

Multisensory training delivered in telerehabilitation is feasible and effective for ameliorating oculomotor compensation of visual field loss, improving mood and reducing functional disabilities in adults with chronic HVFDs.

*Trial registration* This study was retrospectively registered at clinicaltrials.gov (NCT06341777; 26/03/2024).

**Supplementary Information:**

The online version contains supplementary material available at 10.1186/s12984-025-01573-4.

## Introduction

Homonymous visual field defects (HVFDs) are common sequelae of post-chiasmal brain lesions of different etiologies, such as stroke, traumatic brain injury or brain tumors. The rate of spontaneous recovery is maximal within the first months after the brain injury, then the chance of regaining visual functions diminishes over time [1, 2], resulting in chronic visual impairments that compromise vision-dependent activities of daily living (v-ADLs) [3], such as reading and navigating the environment [4–6], with a significant reduction in quality of life [7]. Moreover, individuals with visual loss often experience mental stress and anxiety, which can eventually lead to depression and social isolation [8, 9].

There are different treatment options for HVFDs [10], including compensatory trainings aimed at developing oculomotor strategies to overcome the visual field loss [11–13]. Among compensatory trainings, the multisensory audio-visual training (AVT) [14–21] has been proven effective in promoting the development of efficient oculomotor strategies to compensate for the visual field loss in both adults and children with acquired brain injury [22]. The advantage of AVT, as compared with standard unimodal visual trainings [16], would rely on the activation of a multisensory retino-colliculo-extrastriate pathway that is frequently spared in the case of HVFDs following posterior brain damages [23–26], as also shown in animal models [27, 28].

However, AVT is a rather intensive in-person treatment (requiring several hours of training) that is typically delivered daily on an outpatient basis for two or more weeks [14]. This poses logistical difficulties, forcing some people to forego embarking on the rehabilitation pathway. This is especially true in the chronic phase of the disease, when expectations for improvement are reduced and the commitments of daily life become incompatible with an intensive outpatient treatment. These limits of ambulatory rehabilitation can be overcome with telerehabilitation [29–31], which allows for the remote administration of therapies, hence increasing their accessibility and retaining the person's autonomy. However, the feasibility and effectiveness of remote-supervised, home-based, AVT for acquired HVFDs still need to be documented. Attempts in this direction have been made by Tinelli and colleagues [19], who provided some preliminary evidence of the feasibility of a 5-week home-based AVT for HVFDs but on an individual basis. Similarly, Daibert-Nido et al. [32] developed a home-based virtual-reality protocol for audio-visual stimulation, testing its feasibility in two persons with hemianopia following a pediatric brain tumor (see also [33], for a home-based, but not remotely assisted, 'unimodal' reading and exploration computer training).

Therefore, the present study aimed to explore the feasibility and clinical efficacy of the AVT delivered at home in telerehabilitation in a sample of adults with chronic acquired HVFDs, assessing its effects on oculomotor visual field scanning (primary outcome), reading, and v-ADLs, also characterizing behavioral, neuro-ophthalmological and neuroradiological correlates of treatment-induced clinical gains. Moreover, we also explored the impact of the treatment on mood: if the AVT has a positive impact on visual field exploration, and this in turn improves the quality of life, the expectation is that mood will also benefit. Finally, in a second study, we compared the efficacy of the remotely delivered AVT with that of its in-person version.

## Study 1: Clinical efficacy of telerehabilitation for visual field defects

### Methods

#### Experimental design

This is a single-arm, prospective, evaluator-blinded, clinical trial. All participants underwent in-hospital neuropsychological and neuro-ophthalmological evaluations at the following timepoints: before the beginning of the AVT (Pre), immediately at its end (Post), at 1 month (FU1) and 6 months follow-ups (FU6). Structural Magnetic Resonance Imaging (MRI) was acquired only pre- and post-treatment.

A sub-group of these participants (N = 13; i.e., Waitlist Group, WL) was randomly assigned (1:1 ratio; random sequence generation, enrollment, and allocation carried out by C.C.) to undergo an additional baseline assessment one month prior to the AVT (i.e., Pre -1). This double-baseline approach aimed at controlling for practice effect and spontaneous changes, although unlike in chronic conditions. For more details, see the Supplementary Material.

#### Participants and sample size estimation

**Fig. 1:**
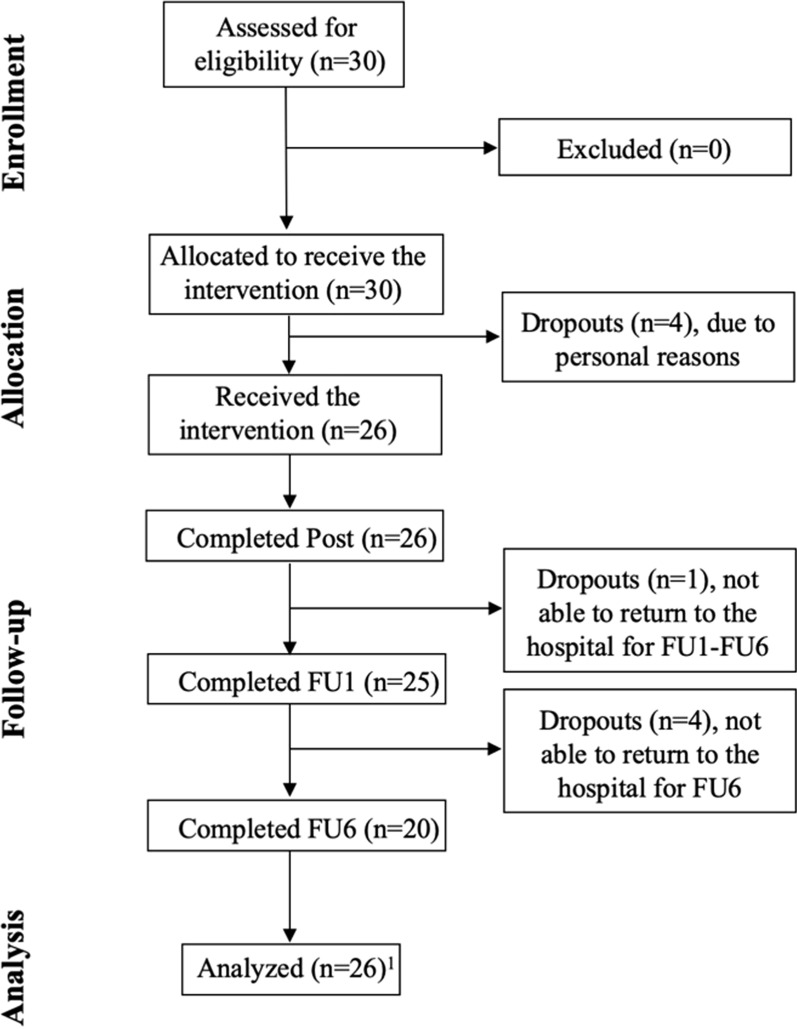
Consort flow diagram. Post: assessment immediately after the treatment, FU1: 1-month follow-up; FU6: 6-month follow-up. ^1^all participants who completed, at least, the Post assessment were included in the analyses

The sample size was calculated with G*Power 3 (Heinrich-Heine-Universität Düsseldorf, Germany), considering changes in visual search performance (primary outcome) [14, 18] in a repeated measures Analysis of Variance (ANOVA) with alpha = 0.05, 1-beta = 0.95, a medium effect size (f = 0.3), 4 timepoints (Pre, Post, FU1, FU6), correlation between repeated measures of 0.5, and sphericity correction = 1. The calculation indicated a minimum sample of 26 adults. Estimating a conservative attrition rate of 15%, we recruited 30 participants (Fig. 1) at the Capitanio Hospital of the IRCCS Istituto Auxologico Italiano (Milan, Italy). Of the 30 recruited participants, 26 completed the training and the Post-training assessment, 25 completed FU1 and 20 completed FU6 (see Fig. 1). A final sample of 26 participants was analyzed (see Table 1), i.e., all participants who completed at least the Post assessment.

**Table 1:** Clinical-demographic characteristics of the analyzed sample (n = 26)

Age (years)	54.2 ± 16.3
Sex	14 females, 12 males
Disease duration (month)	28.7 ± 25
Etiology	10 hemorrhagic stroke 12 ischemic stroke 3 TBI 1 brain tumor
Visual field size (MD, db)	− 12.6 ± 4.4
Visual field defect	2 left inf. quad. 13 left HH 11 right HH
Lesion volume (cm^3^)	29 ± 33.1
EF test—accuracy (%)	86.1 ± 17.2
Triangles test—accuracy (%)	78.4 ± 14.1
Numbers test—RTs (ms)	34.37 ± 10,218
Reading speed (syllables/sec.)	3.8 ± 1.6
v-ADLs	10.3 ± 6.39
HDRS	7.1 ± 5.1

*N*europsychological and neuro-ophthalmological results refer to the baseline assessment before the beginning of the training, i.e., Pre. For quantitative variables, mean ± standard deviation is reported

MD, mean deviation in decibels (db) at 30–2 visual field testing; inf. quad., inferior quadrantanopia; TBI, traumatic brain injury; HH, homonymous hemianopia; RTs, response times; v-ADLs, vision-dependent activities of daily living; HDRS, Hamilton Depression Rating Scale

**Fig. 2:**
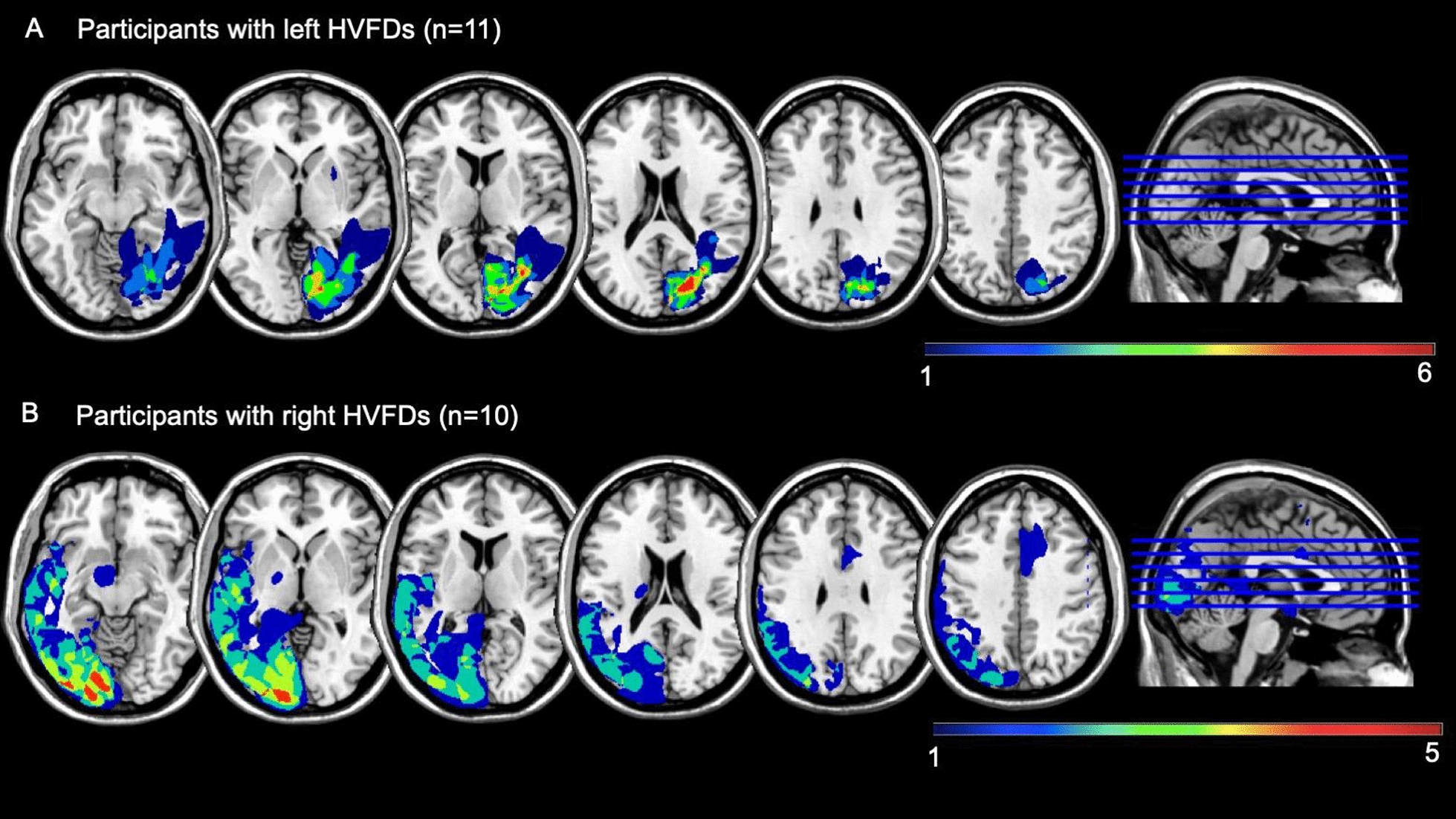
Lesion localization of participants with homonymous visual field defects (HVFDs). Overlay lesion plots (frequencies of overlapping lesions, from dark blue, i.e., minimum overlap, to red, i.e., maximum overlap) for A) participants with left HVFDs and B) participants with right HVFDs. Brain lesions are displayed according to the neurological convention. The average lesion volume was 29 ± 33 cm^3^ (range: 0.3–114 cm^3^). According to the AAL atlas, the most affected areas, irrespective of the lesion side, were: the calcarine sulcus (N = 15), the lingual gyrus (N = 15), the superior (N = 9), the middle (N = 12), and the inferior (N = 9) occipital lobes, as well as the cuneus (N = 13), and the fusiform gyrus (N = 12). In fact, the most injured lobes were the occipital lobe (N = 16; mean lesion extension = 15.53 ± 16.05 voxels), followed by temporo-parietal areas (N = 13; mean lesion extension = 13.58 ± 20.84 voxels). Only few participants experienced damages to the frontal lobe (N = 3) or the sub-cortical nuclei (N = 4)

Inclusion criteria were: age > 18 years, acquired chronic HVFDs (disease duration ≥ 6 months, confirmed by visual field perimetry) due to stroke, traumatic brain injury, or brain tumor; absence of cognitive decline and major psychiatric/neurological diseases (as assessed through medical history and clinical interview at the screening phase, or by considering the neuropsychological assessment whenever available); normal hearing; normal visual acuity (best corrected monocular visual acuity = 20/20, assessed with Snellen chart); no history or evidence of retinal disease, retinal surgery, ocular trauma, optic neuropathies (including glaucoma and ocular hypertension), myopia equal to or greater than 6 diopters; not being enrolled in another treatment for HVFDs. The reconstruction of brain lesions is depicted in Fig. 2.

#### Home-based AVT

**Fig. 3:**
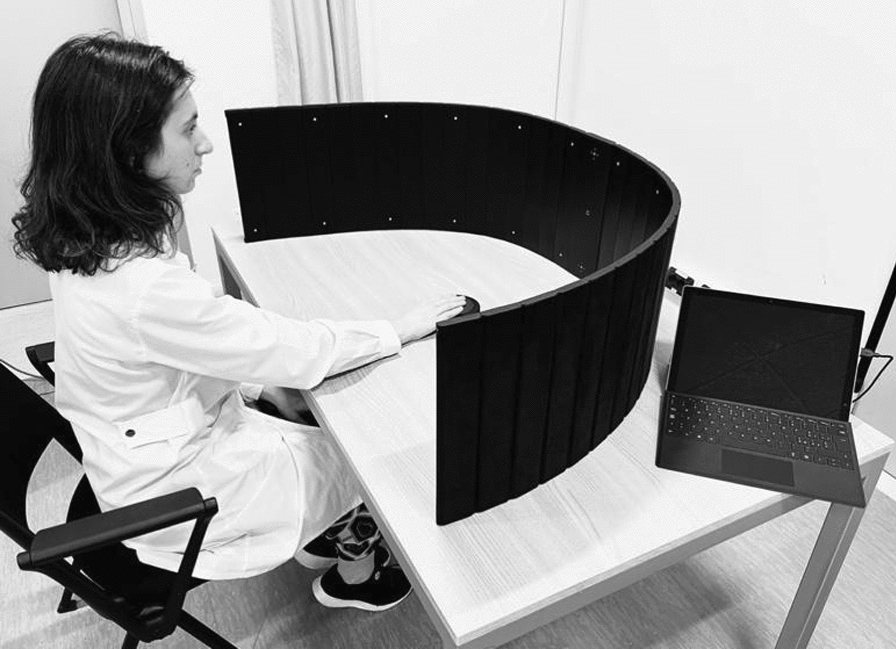
AvDesk setup for home-based audio-visual training

The AVT was delivered via the AvDesk device (Linari Medical; https://linarimedical.com), a foldable, semicircular panel (length 192 cm; diameter 110 cm; see Fig. 3) that features 24 visual units (light emitting diodes, LEDs) arranged in two horizontal rows (12 units for each row), and 12 audio units (loudspeakers arranged in one horizontal row) at an eccentricity of 8, 24, 40, 56, 72, 88 degrees from the central fixation point, in the left and in the right hemifield. An eye- and face-tracking camera in the center of the panel monitored the gaze position: a stimulus was presented only if the central fixation was maintained. The camera had a sampling rate of 10 Hz and it was integrated with a self-developed software that enables head detection with an accuracy of 1 mm in the x and y planes of the capture area, as well as gaze and head direction with an accuracy of 1°. During the AVT, the participant sat in front of the AvDesk at a distance of ~ 57 cm, so that the panel covered 180° of visual field. Participants were instructed to orient their gaze towards the audio-visual stimulus (a red flash simultaneous with a sound at 2800 Hz; duration = 100 ms) and press a wireless button as soon as the visual stimulus was detected. To monitor false positives, catch trials (15% of auditory stimuli, without visual ones) were presented.

Participants were required to train at any time of the day, for 2 h, 5 days a week for 3 weeks. Breaks were allowed during daily treatment, which was organized into two or more blocks of trials according to the participant’s needs. The total number of daily stimuli varied depending on the individual performance and progression, and in every session, stimuli were presented in random order in the blind visual field (70%) and in the intact visual field (30%).

At the end of each session, accuracy (i.e., the percentage of correct audio-visual detections via button press) and response times (RTs) were automatically sent to an online server accessed by the therapist to remotely monitor the treatment progress. Data connection was always guaranteed through a data SIM card installed in the AvDesk computer or via connection to the participant’s home network (Ethernet cable or Wi-Fi).

Before the beginning of the training, participants and their caregivers underwent an in-person training providing detailed instructions on the use of the AvDesk. Remote supervision and assistance during the training was possible thanks to the server-based system, that allowed the therapist to monitor daily adherence to treatment schedule.

### Neuropsychological assessment

#### Visual search

Three different tasks were used to assess visual field search: the EF, Triangles and Numbers tests [14, 21]. For all tests, the stimuli were presented on an LCD screen (Philips LCD 42″; resolution: 1920 × 1080), at a distance of 57 cm from the eyes (visual angle = 79° horizontal × 48° vertical). Each trial began with a fixation (a red cross lasting 1 s), followed by the presentation of the search array on a black background. Participants had to scan the visual field and look for visual targets presented among distractors of the same size. Participants were instructed to respond as accurately and quickly as possible. After the response, a black screen was presented for 1 s before the start of the next trial. The experimenter ensured by visual inspection that the participant looked at the fixation cross before starting the next trial.

In the EF test, the arrays contained 21 randomly distributed stimuli (5 × 5 cm green letters): the target letter ‘F’ among the distractor letters ‘E’. Participants had to scan the array and indicate the presence of the target by pressing the left arrow key of the PC keyboard or its absence by pressing the right arrow key. Twenty trials were presented: 16 with the target and 4 without. In each trial, the target was located in one of 16 different areas of the screen, i.e., the screen was ideally subdivided into four columns and four rows, resulting in 16 rectangular areas.

In the Triangles test, each array comprised 21 visual stimuli (4 × 4 cm): targets were yellow triangles (0 to 13) and distractors were yellow squares (target/distractor ratio: 0–62%). The participant had to scan the visual array and verbally report the number of targets, pressing the space bar of the keyboard to indicate the end of the scan. Twenty trials were administered.

In the Numbers test, eight arrays of stimuli were presented in random order, each containing 15 red numbers (from number 1 to 15): the task was to point, with the dominant hand, to each number in ascending order as fast as possible. Each trial was terminated by the experimenter (button press of a keyboard) when the participant correctly pointed to all the 15 numbers. The score was the mean RT needed to search for all the numbers.

Accuracy was recorded for the EF and the Triangles tests (i.e., the ratio between the number of correct responses and the total number of trials, in percentage), and median RTs for all the three visual search tests. Stimuli presentation and response recording was controlled by the E-Prime 2 software (Psychology Software Tools Inc., Pittsburgh, PA).

#### Reading

The reading test consisted of a short story (“Il terzo mondo”) [34]. Reading speed (number of syllables/s) and the number of errors corrected for the number of read syllables were calculated. One point was assigned for each reading error (i.e., omissions, substitutions of syllables of words), reading the wrong line or reading latencies > 5 s; 0.5 points were attributed for self-corrections.

#### v-ADLs

We administered a 10-item questionnaire [14, 35] assessing the most frequent visual difficulties experienced by people with HVFDs. Participants had to rate on a 5-point scale (from 0 = no problem to 4 = very frequent and relevant problem) to what extent they experienced difficulties in performing the following activities: noticing obstacles, bumping into objects, losing the way, finding objects on a table, in a room, or in the supermarket, walking in a crowd, reading, going up and down the stairs, and crossing the street. The total score range was 0–40.

#### Mood

Depressive and anxiety symptoms were assessed with the Hamilton Depression Rating Scale (HDRS) [36]. The scale is composed of 21 items covering typical symptoms of depression (depressed mood, sense of guilt, suicidal thoughts, sleep disturbances, changes in weight, anxiety, and paranoid or obsessive thoughts), rated on a 5-points scale (1 = absent symptom to 5 = severe symptom). Scores are summed and the severity of depression is scored as follows: 10–15 (possible depression), 16–25 (mild depression), 26–28 (moderate depression), > 28 (severe depression).

#### Multisensory integration

Multisensory, audio-visual, integration ability was assessed with the Simultaneity audio-visual Judgement task (SJ2) [37], which allows to measure the so-called temporal binding window (TBW) [38, 39]. In the SJ2 task, participants are asked to judge the simultaneity of an auditory stimulus (a pure tone of 3500 Hz; duration = 30 ms) and a visual stimulus (a white ring on a black background; diameter = 9.4 cm, duration = 30 ms) presented together, by pressing the upward arrow key if they are perceived simultaneous or the downward arrow key if not. Inter-trial interval ranges from 2 to 3 s. During the task, auditory and visual stimuli were presented with a stimulus onset asynchronies (SOA) of 0, ± 50, ± 100, ± 150, ± 200, ± 250, ± 300, ± 350, ± 400 ms (- means auditory first, + visual first). For each SOA, 20 trials were given, for a total of 340 trials presented in random order. Stimulus presentation and response recording were controlled by the E-Prime 2 software. For details, see [38].

### Neuro-ophthalmological assessment

#### Visual field perimetry

HVFDs were measured with a Humphrey’s field analyzer. Monocular assessment was performed for both eyes, applying a Swedish interactive thresholding algorithm (SITA standard), testing the central 30° (30–2) of visual field. Mean Deviation (MD) values of both eyes were averaged and used for the analyses. Negative values reflect a deviation from the expected performance in the participant’s age group, hence a visual field defect.

#### Visual evoked potentials (VEPs)

Pattern-reversal VEPs were recorded with a 4-recording channels computerized system (RETIMAX, CSO, IT) at Pre, Post, and FU6. We presented pattern-shift stimuli, using a black-and-white checkerboards displayed on a CRT screen (contrast 100%, luminance 10 cd/m^2^, frequency = 1 Hz, pattern size = 15’).

Recording was performed during monocular hemifield stimulation to analyze evoked patterns from both the sighted and the healthy hemifield. Specifically, cortical VEPs were recorded through surface electrodes positioned over OZ (active electrode), FZ (reference), and the mastoid (ground). The following recording parameters were employed: pass-band from 1 to 30 Hz, acquisition time 300 ms, at least 100 averages.

We analyzed latency and peak amplitude of the P100, the most stable and repeatable component, averaging values of the monocular recordings for the 15’ checkboard.

### Neuroimaging: lesion mapping and structural connectivity

Structural Magnetic Resonance Imaging (MRI) sequences were acquired in two 3 T MRI scanners, a Philips Ingenia CX (Fondazione IRCCS San Gerardo dei Tintori, Monza, Italy) and a GE Signa Premier (IRCCS Istituto Auxologico Italiano, Milan, Italy). Each participant performed the pre- and post-training examination at the same location, thus with the same scanner.

Both hospitals shared the same acquisition protocol that included: a 3D high-resolution T1-weighted sequence (FSPGR, TR/TE = 10.7/4.9 ms, flip angle = 13, 1 mm^3^ isotropic voxels)**,** and a diffusion-weighted multi-shell sequence (DWI-EPI, 66 directions gradient with a maximum b-value = 2000s/mm2, TR/TE = 17,000/89.4 ms, 2.6 mm^3^ isotropic voxels). Moreover, a b = 0 sequence with reverse phase-encoded direction was also acquired to correct for geometric distortions. The Neuroradiologists involved in the study confirmed that the imaging outputs from the two scanners had comparable quality.

From MRI scans, brain lesions were manually reconstructed on T1 scans in MRIcroGL [40]. Brain scans and lesion maps were normalized onto an age-appropriate template by means of the “MR segment-normalize” function of the Clinical Toolbox [41] for Statistical Parametric Mapping (SPM12) [42], in MATLAB 2019b (The MathWorks Inc., 2019). We extracted the mean lesion volume (cm^3^) and the lesion extension (i.e., number of voxels) within cerebral areas according to the Automated Anatomical Labelling atlas (AAL) [43]. Moreover, we calculated the overall lesion extension of the occipital, temporal, and parietal lobes by summing the lesion extension of single areas [21, 44]. Pre-processing of DTI data was performed with FSL. Diffusion-weighted images were first corrected for movement artefacts, physiological noise, geometric distortions, and eddy currents. The obtained images were co-registered with the anatomical ones, applying a rigid transformation. By using MRtrix, we obtained Fractional Anisotropy (FA) and Mean Diffusivity anisotropy (MDiff) maps; white matter tracts were then reconstructed. Here, we applied a probabilistic algorithm based on Constrained Spherical Deconvolution (CSD) [45–47], selecting a maximum of 1000 tracts. Subsequently, regions of interests were delineated on T1 scans to identify tracts following anatomical landmarks: the cortico-spinal tract (CST), chosen as a reference tract since it is usually spared in people with HFVDs, the inferior longitudinal fasciculus (ILF), the superior longitudinal fasciculus (SLF), the inferior fronto-occipital fasciculus (IFOF), the optic radiations (OR), and the optic tract (OT). FA and MDiff were calculated for all tracts in both hemispheres.

### Statistical analysis

Analyses were performed with jamovi 2.4.14 [48]. α was set at 0.05.

#### Baseline performance stability

In order to assess the stability of deficits before the treatment, we performed Wilcoxon tests comparing the two baselines (Pre-1 and Pre) of the WL group, in terms of visual search tests performance (Accuracy and RTs), reading performance, functional burden in the ADLs, depressive symptoms, and visual field size.

#### Treatment effects

All participants (n = 26) were included in the analyses. To determine treatment’s effects (i.e., changes in accuracy during the training, as well as post-training changes in neuropsychological, neuro-ophthalmological, and neuroimaging variables; see below), different mixed models were used depending on distribution of residuals, by Q-Q plot inspection and Kolmogorov–Smirnov test of normality. Linear Mixed Models (LMMs) were used in case of normally or quasi-normally distributed residuals, whereas generalized mixed models (GMMs) fitting gamma distribution were used in case of or severely skewed data [44, 49]. For LMMs, degrees of freedom and *p*-values were calculated with the Satterthwaite method. For all models, random intercepts were calculated for participants and significant interactions were explored with Holm-corrected post-hocs.

#### Audio-visual detections during the training

To assess the improvements during the 3 weeks of training, we analyzed accuracy changes in the detection of audio-visual stimuli (i.e., the percentage of correct audio-visual detections) by comparing accuracy on Day 1 and Day 15 of the training. A GMM was run with Day (Day 1 and Day 15) and Hemifield (Blind and Sighted) as within-subject fixed factors. The accuracy on catch trials was analyzed by means of a GMM with Day as within-subject factor.

#### Neuropsychological outcomes

Median accuracy in visual search were analyzed for the EF and the Triangles tests by means of an LMM with Timepoint (Pre, Post, FU1, and FU6) and Test (EF and Triangles) as within-subject fixed factors. Changes in visual search speed were analyzed considering median RTs in the Numbers test, which were used as dependent variable in a LMM with Timepoint as fixed factor (see the Supplementary Material for the RTs analysis of the EF and the Triangles tests). Reading performance (i.e., the number of errors and reading speed in syllables/s), as well as the total scores of the v-ADLs questionnaire and the HDRS scale were entered as dependent variables in separate mixed-models with Timepoint as fixed factor. As for the measure of multisensory integration (i.e., the amplitude of the TBW), we performed a Wilcoxon test between the Pre and the Post values.

#### Neuro-ophthalmological outcomes

To investigate changes in visual field size, we ran an LMM on the MD values with Timepoint (Pre, Post, FU1, and FU6) as fixed factor. The amplitude and latency of the P100 component of PEVs were entered as dependent variables in two different LMMs, with fixed factors Timepoint (Pre, Post, and FU6) and Hemifield (i.e., blinded and sighted).

#### Neuroimaging data

Post-treatment changes in MDiff and FA of white-matter tracts in the lesioned hemisphere were analyzed by means of LMMs with Timepoint (Pre and Post) and Tract (CST, ILF, SLF, IFOF, OR, and OT) as within-subject fixed factors.

#### Predictors of AVT efficacy

To reveal possible characteristics associated with AVT clinical benefits, Spearman correlations were used to assess significant correlations between AVT-induced improvement in visual search accuracy (EF and Triangles test), speed (Numbers test), and v-ADLs (the average of post-treatment scores *minus* baseline scores, ΔPost-Pre) with different variables collected at baseline. These included: clinical-demographic variables (age and disease duration), baseline performance on visual search and the v-ADLs questionnaire, neuro-ophthalmological (MD in 30–2 assessment, amplitude, and latency of the P100 evoked from the blind hemifield) and neuroimaging data (total lesion volume, extension of occipital, temporal, and parietal lobe damage, as well as FA and MDiff of the lesioned hemisphere tracts).

## Results

### Baseline performance stability

No significant changes were observed between baselines in the WL group, with a small trend for the EF test (all *Ws* > 6, all *ps* > 0.05; Table 2).

**Table 2:** Baseline stability in the wait-list (WL) group

Variable	Pre-1	Pre	Comparison
EF test–accuracy	83.4 ± 16.7%	88.3 ± 12.2%	*p* = 0.06
Triangles test–accuracy	72.6 ± 15.4%	77.2 ± 15.4%	*p* = 0.17
Numbers Test–RTs (ms)	35.17 ± 10.47	34.08 ± 12.28	*p* = 0.31
Reading speed (syll/s)	3.6 ± 1.5	3.6 ± 1.5	*p* = 0.99
Visual field size (MD, db)	− 13.2 ± 4	− 14 ± 2.7	*p* = 0.15
v-ADLs	9.5 ± 4.6	9.2 ± 4.6	*p* = 0.47
HDRS	7.7 ± 6.8	7 ± 6	*p* = 0.48

For the visual exploration tests (EF, Triangles, and Numbers), the visual field assessment, and the vision-dependent activities of daily living (v-ADLs) questionnaire, n = 13 participants were analyzed. Twelve participants completed the Hamilton Depression Rating Scale (HDRS) and 10 participants completed the reading test. Those participants who could not complete the latter tests had reading impairments due to left-hemispheric lesion

MD, mean deviation in decibels at 30–2 visual field testing; Pre, baseline assessment immediately before the beginning of treatment; Pre-1, additional baseline assessment one month before the beginning of the treatment; RTs, response times

### Audio-visual detections during the training

The GMM analyses showed only a main effect of Hemifield (*X*^*2*^ = 41.37, *p* < 0.001), with overall lower accuracies in the blind hemifield. Effects of Day (*X*^*2*^ = 0.58, *p* = 0.45) and Day*Hemifield interaction (*X*^*2*^ = 0.26, *p* = 0.61) did not attain the significance level, showing only a mild global improvement during the training (Day 1, mean ± Standard Error = 89.5% ± 2.2% vs. Day 15, end of the 3rd week of AVT = 91.4% ± 2.2%).

Catch trials rate was low (6% ± 7.8%) and stable during the training (Day: *X*^*2*^ = 1.94, *p* = 0.38), showing that participants, overall, did not press the button in absence of visual stimuli.

### Neuropsychological outcomes

**Fig. 4:**
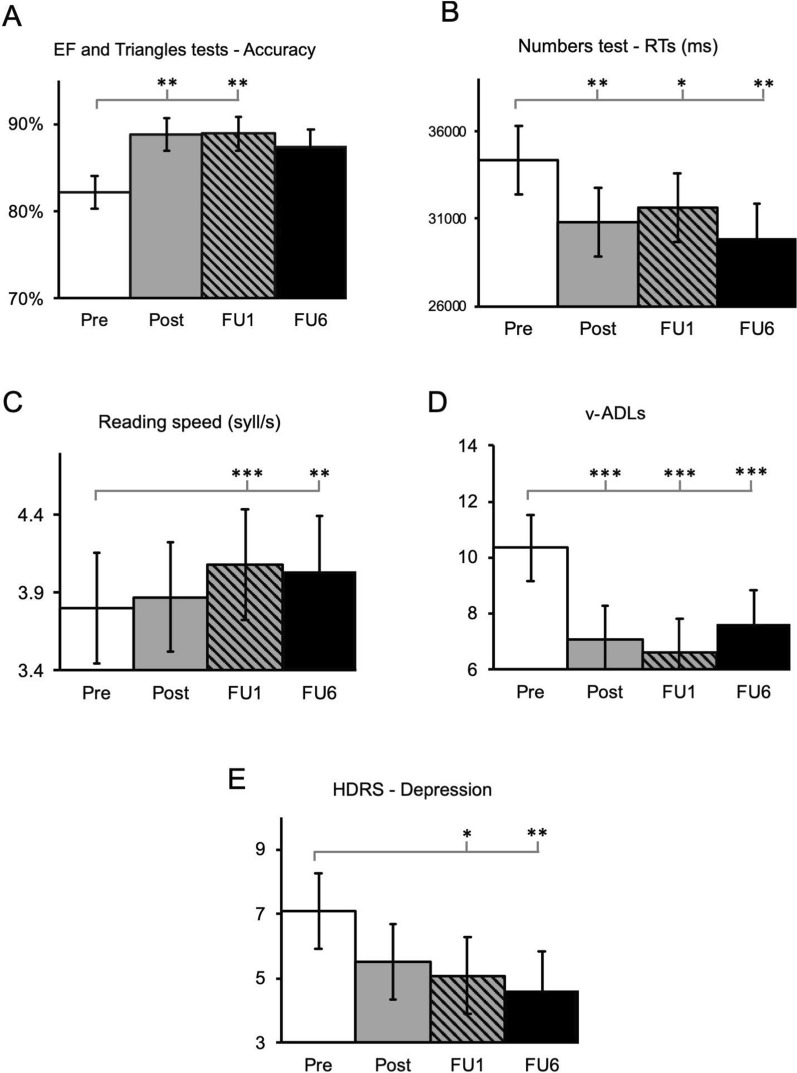
Changes in neuropsychological measures. All participants (n = 26) were included in the analysis. Error bars depict the Standard Error (SE). RTs, response times; v-ADLs, vision-dependent activities of daily living; HDRS, Hamilton Depression Rating Scale. Pre, baseline values, before the treatment; Post, assessment immediately after the treatment, FU1, 1-month follow-up; FU6 = 6-month follow-up. Comparisons with baseline (Pre): *p < 0.05, **p < 0.01, ***p < 0.001

Regarding the accuracy in the visual search tests (EF and Triangles tests, see Fig. 4), the LMM analyses revealed a significant effect of Timepoint (*F*_*3,163*_ = 5.33, *p* = 0.002), showing an improvement from baseline (82.2% ± 1.9%) to post-treatments up to 1-month FU (Post = 88.8% ± 1.9%, *p* = 0.004; FU1 = 88.9% ± 1.9%, *p* = 0.004); from FU1 to the 6-months FU, performance remained stable (*p* = 0.99). We also observed an effect of Test (*F*_*1,161*_ = 30.47, *p* < 0.001), indicating overall higher accuracies in the EF test (90.7% ± 1.7%) than in the Triangles test (83% ± 1.7%). The interaction was not significant (*F*_*3,161*_ = 0.15, *p* = 0.93).

A speed-up of visual search (RTs) emerged in the Numbers test, as shown by the significant effect of Timepoint (*F*_3,68.3_ = 7.07, *p* < 0.001). Compared to pre-treatment (34 s ± 2.5 s) participants showed faster exploration times immediately after the treatment (30.7 s ± 2.5 s, *p* = 0.003), at FU1 (31.6 s ± 2 s, *p* = 0.035), and up to the 6-month follow-up (29.8 s ± 2 s, *p* < 0.001).

In the reading test, the LMM analysis revealed an effect Timepoint (*F*_3,55_ = 7.09, *p* < 0.001), showing an improvement of reading time at 1-month (4.08 ± 0.4 syllable/s, *p* < 0.001) and the 6-month follow-ups (4.05 ± 0.4 syllable/s, *p* = 0.015), as compared to baseline (3.8 ± 0.4 syllable/s). In terms of errors, the main effect of Timepoint (*X*^*2*^ = 9.4, *p* = 0.025) showed improved scores at the follow-up at 6 months only (21.9 ± 4.1, *p* = 0.033), compared to the baseline performance (24.2 ± 4.1).

With respect to v-ADLs, a significant reduction of vision-related burden was found (*F*_3,68.2_ = 10.7, *p* < 0.001): compared to pre-treatment scores (10.4 ± 1.2) participants experienced a reduced visual-related disability immediately at the end of the treatment (7.1 ± 1.2, *p* < 0.001), stable at the follow-ups (FU1 = 6.6 ± 1.2, *p* < 0.001, FU6 = 7.6 ± 1.2, *p* = 0.004).

Finally, the LMM of the HDRS revealed a reduction of depressive symptoms (*F*_3,61.7_ = 4.81, *p* = 0.004) at FU1 (5.1 ± 0.9, *p* = 0.02) and FU6 (4.62 ± 0.9, *p* = 0.007), compared to the baseline scores (7.1 ± 0.9).

As for the multisensory integration, the amplitude of the temporal binding window was calculated for n = 20, i.e., those who completed the task both before and after the treatment. The Wilcoxon test did not yield significant pre-post differences (*W* = 146, *p* = 0.133).

### Neuro-ophthalmological outcomes

The LMM analysis of the MD values from visual field perimetry showed an effect of Timepoint (*F*_3,68.2_ = 3.32, p = 0.025), suggesting an improvement in visual field size that however did not survive the multiple comparisons (all *ps* > 0.07).

The LMMs of VEPs showed no significant effects of Timepoint on P100 latency (*F*_2,115_ = 0.98, *p* = 0.38) and an effect on the amplitude (*F*_2,115_ = 3.3, *p* = 0.04), suggesting a decrease immediately after the treatment (Post: 6 ± 0.5 µV; *p* = 0.052), with no changes at FU6 (6.2 ± 0.5 µV; *p* = 0.14), as compared to the baseline (Pre: 7 ± 0.5 µV). An effect of Hemifield was also observed both for the amplitude (*F*_1,114_ = 44.18, *p* < 0.001) and the latency (*F*_1,111_ = 11.68, *p* < 0.001), indicating reduced amplitude and increased latency in the blind compared to the sighted hemifield. No significant interactions were observed (all *Fs* < 2.2, all *ps* > 0.11).

### Neuroimaging data

Four participants could not undergo the MRI procedures, because of counterindications. Moreover, tracts could be reconstructed for 21 participants, whose lesion pattern allowed the computation of FA and MDiff.

The LMM analyses of white-matter structural connectivity metrics revealed no effects of Timepoint (FA: *F*_1,210_ = 1.37, *p* = 0.24; MDiff: *F*_1,210_ = 1.51, *p* = 0.22) or Timepoint*Tract interaction (FA: *F*_5,210_ = 0.24, *p* = 0.95; MDiff: *F*_5,210_ = 0.12, *p* = 0.99). Nonetheless, we observed differences in FA between tracts (*F*_5,210_ = 23.11, *p* < 0.22): compared to the control tract CST (0.3 ± 0.01), lower FA was observed in all other tracts relevant to visuospatial functions (all *ps* < 0.007): the IFOF (0.24 ± 0.01), the ILF (0.18 ± 0.01), the OR (0.26 ± 0.01), the OT (0.25 ± 0.01), and the SLF (0.24 ± 0.01). Moreover, lower FA was detected in the ILF, as compared to OR, OT, and SLF (all *ps* < 0.001). A similar effect of Tract was observed for the MDiff values (*F*_5,210_ = 15.60, *p* < 0.001), with the IFOF (0.73 ± 0.03), the ILF (0.79 ± 0.03), the OR (0.8 ± 0.03), the OT (0.82 ± 0.03), and the SLF (0.74 ± 0.03) showing higher MDiffs compared to the CST (0.67 ± 0.03; all *ps* < 0.008). Finally, MDiff of the IFOF was lower as compared to the ILF, the OT, the OR (all *ps* < 0.009), as well as lower values of the SLF compared to the OT, the OR, and the ILF (all *ps* < 0.043).

### Predictors of AVT efficacy

Spearman correlations showed a negative association between baseline performances and the respective post-treatment improvements (i.e., calculated as Δ Post *minus* Pre scores). This association emerged when considering the accuracy in the EF (*rs* =—0.76, *p* < 0.001), and the Triangles (*rs* =—0.62, *p* < 0.001) tests, and v-ADL scores (*rs* =—0.54, *p* = 0.005): larger gains were present in participants who were more impaired before the AVT. Moreover, improvements in visual search accuracy were larger in those participants with more impaired functionality of the visual pathways, namely, those presenting with longer P100 latency (Triangles test; *rs* =—0.5, *p* = 0.01) and smaller P100 amplitude (EF test; *rs* =—0.45, *p* = 0.02). We also observed an effect of multisensory integration abilities, i.e., a narrower TBW (reflecting better multisensory integration) was associated to larger improvements in the EF test (EF test; *rs* =—0.49, *p* = 0.03). Finally, post-treatment improvements in visual search speed (RTs in the Numbers test) were larger in those participants with reduced structural connectivity of the OR (MDiff; *rs* = 0.57, *p* = 0.008). No other significant correlations emerged (all *rs* < 0.47, all *ps* > 0.06; all correlations are reported in Table S2 of the Supplementary Material).

## Study 2: Comparison of home-based and in-person versions of the AVT

The behavioral effects of the home-based AVT were compared to those of a 2-week in-person outpatient AVT (IP group) carried out by a different sample of participants with chronic HVFDs (N = 16; 11 males and 5 females; mean age = 50 ± 13 years; mean disease duration = 454.2 ± 353 days; 9 ischemic strokes, 7 hemorrhagic strokes; 10 right hemianopias, 4 left hemianopias and 1 left inferior quadrantanopia). These data, which have never been published, were collected in the context of specialization thesis work of C.C., for which participants gave their informed consent.

For the home-based AVT (HB, from now on), we selected a sub-group of participants from the main experiment (i.e., those who started the treatment immediately after the Pre evaluation; n = 13) because they performed the assessment with the very same timeline as the IP group (i.e., Pre, Post, FU1, and FU6). As outcome measures, we considered scores on EF, Triangles, and Number tests, and v-ADLs questionnaire.

The in-person, hospital-based, AVT consisted in the detection of audio-visual stimuli delivered on a training board (central 1 × 2 m part, with two 2 × 0.5 m side wings tilted 45° inward) with 48 red LEDs, diameter = 1 cm, luminance = 90 cd m^2^) arranged in six horizontal rows (eight lights per row), distributed on the board; 48 piezoelectric speakers (0.4 W, 8 Ω; auditory stimulus = 80 dB) were arranged at the LEDs location. Each audio-visual unit was separated by 12° of visual angle, i.e., the panel covered 84° horizontally × 64° vertically (EMS srl, Bologna, Italy; www.emsmedical.net). During the training, spatially and temporally coincident audio-visual stimuli (100 ms) were randomly presented, one at a time, at one of 48 locations (24 locations for each hemifield). The participants were instructed to look at the central fixation point (2°) and move their eyes toward the audio-visual stimulus, indicating the detection of the visual stimulus by pressing the button of a wireless mouse. The experimenter monitored the participants’ gaze throughout the training and started the next trial only after the participant’s eyes returned to the central fixation point; for details, see [14, 21, 44]. The AVT consisted of 10 daily sessions (Monday to Friday) in which blocks of 96 audio-visual trials were administered (two trials per spatial location; average block duration = 10 min). In each session, the number of blocks varied according to the participant's speed or fatigue. Each daily session lasted 2 h.

### Statistical analysis

#### Audio-visual detections during the training

Improvements during the trainings were analyzed by comparing the detection accuracy of audio-visual stimuli (i.e., the percentage of correct audio-visual detections) on the first day of training and the last day of training in the two groups. Thus, an LMM was run with Day (first and last days of AVT), Group (HB and IP), and Hemifield (Blind and Sighted) as fixed factors.

#### Neuropsychological outcomes

Post-treatment effects were analyzed by considering visual search median accuracy (EF and Triangles tests; see the Supplementary Material for the RTs analyses of these tests) and speed (RTs in the Numbers tests), as well as v-ADLs. By means of LMM analyses, we compared changes between the two Groups (HB vs. IP), at different Timepoints (Pre, Post, FU1, and FU6), both fixed factors of the models. The LMM on visual search accuracy also included the fixed factor Test (EF and Triangles test). Finally, for the analysis of the v-ADL questionnaire, we considered only Pre, FU1, and FU6, to match the assessments of the IP group (i.e., v-ADL had not been tested at Post in the IP group, according to the respective research protocol). For all models, degrees of freedom and p-values were calculated with the Satterthwaite method and random intercepts were calculated for participants. Significant interactions were explored with Holm-corrected post-hocs.

## Results

### Audio-visual detections during the training

**Fig. 5:**
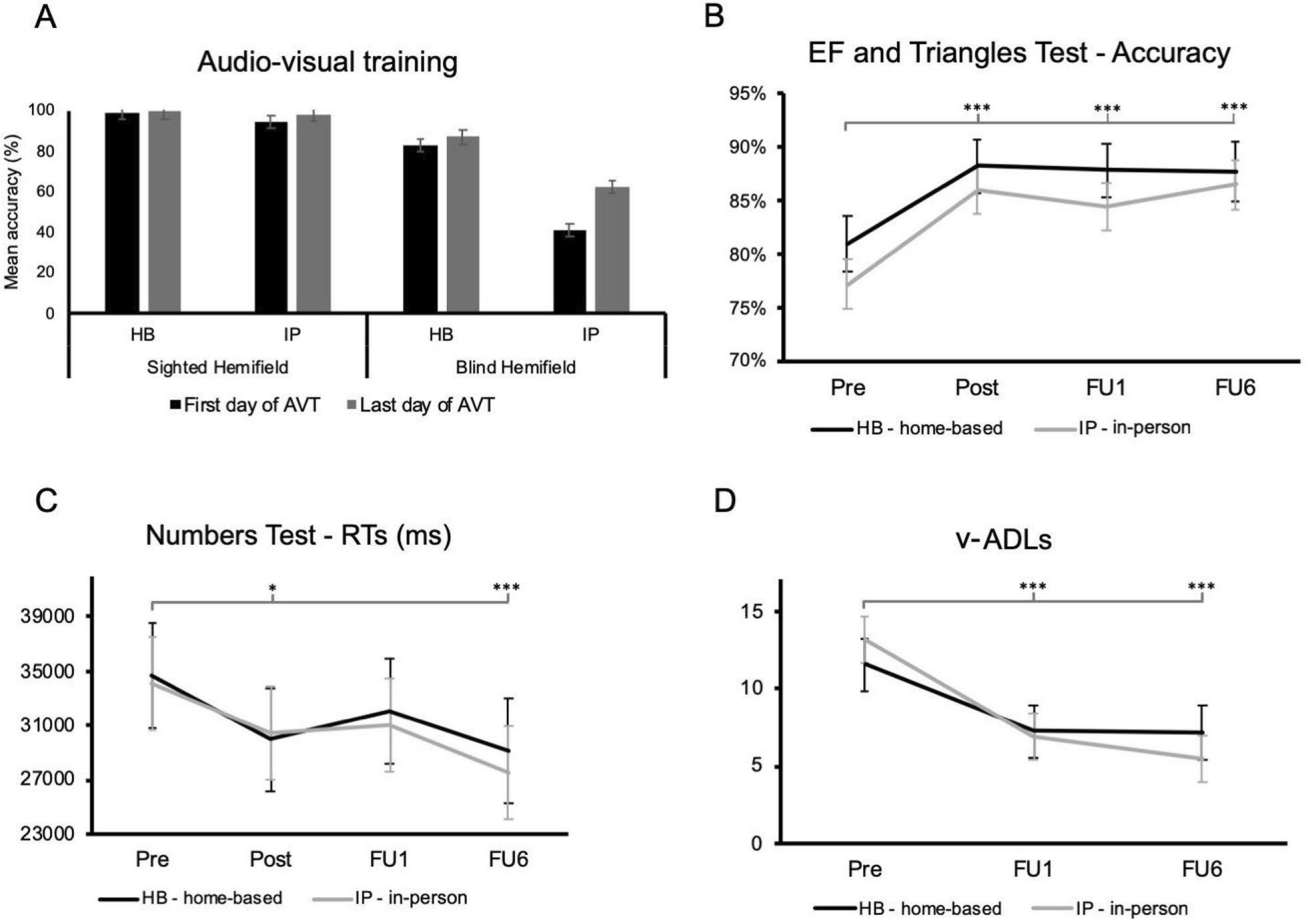
Comparisons of home-based (HB) and in-person (IP) audio-visual trainings (AVT). **A** Audio-visual detection accuracy between the first day of training (in black) and the last day of training (in grey) for both groups and hemifields. Significant post-treatment changes in **B** visual search accuracy, **C** visual search times (RTs), and **D** vision-related activities of daily living (v-ADLs). Black lines represent the HB group, whereas grey lines the IP group. Error bars represent the SE. Pre, baseline values, before the treatment; Post, assessment immediately after the treatment, FU1, 1-month follow-up, FU6 = 6-month follow-up. *p < 0.05, ***p < 0.001

The LMM analysis of audio-visual detections during the trainings (see Fig. 5A) showed a significant effects of Day (*F*_1,81_ = 11.64, *p* < 0.001) and Group (*F*_1,27_ = 43.12, *p* < 0.001), as well as of their interaction (*F*_1,81_ = 5.28, *p* = 0.024): only the IP group, which on the first day of the training showed a worse performance (IP = 40.8% ± 3.1% vs. HB = 82.3% ± 3.5%), significantly improved at the end of the training (difference between the first vs. the last day of training = + 12.3% ± 2.9%; *p* < 0.001), whereas the HB did not (+ 2.4% ± 3.2%; *p* = 0.99). We also found significant effects of Hemifield (*F*_1,27_ = 43.12, *p* < 0.001), Hemifield*Day (*F*_1,81_ = 6.08, *p* = 0.016) and Hemifield*Group (*F*_1,81_ = 48.97, *p* < 0.001). The Group*Hemifield*Day was not significant (*F*_1,81_ = 2.87, *p* = 0.09).

### Neuropsychological outcomes

The analysis of visual search accuracy highlighted the effect of Timepoint (*F*_3, 184_ = 8.62, *p* < 0.001) showing that both groups improved after the training (Post: 87% ± 1.7%; FU1: 86% ± 1.7%; FU6: 87% ± 1.8%; all *ps* < 0.001) as compared to the baseline performance (80% ± 1.7%; see Fig. 5B). The effect of Test was also significant (*F*_1,182.9_ = 33.39, *p* < 0.001, higher accuracies in the EF test), but no effect of Group (*F*_1,27.1_ = 1.17, *p* = 0.29) or Test*Group interaction (all *Fs* < 1.17, all *ps* > 0.28) were found.

Similar results emerged in terms of visual search speed (RTs in the Number test), with a significant main effect of Timepoint (*F*_3, 78.2_ = 6.43, *p* < 0.001), no differences between Groups (*F*_1,27.1_ = 0.02, *p* = 0.88) or Timepoint*Group interaction (*F*_3,78.2_ = 0.2, *p* = 0.9): compared to baseline (34.3 s ± 2.6 s), both groups showed faster visual explorations immediately after the treatment (30.2 s ± 2.6 s; *p* = 0.015), and at the follow-up of 6 months (28.3 s ± 2.6 s; *p* < 0.001; see Fig. 5C).

Finally, as indicated by the effect of Timepoint (*F*_2,51.2_ = 27.2, *p* < 0.001), both groups (Group: *F*_1, 26.9_ = 0.001, *p* = 0.95; Group*Timepoint: *F*_2,51.2_ = 1.62, *p* = 0.21) showed a significant reduction of vision-related burden in v-ADLs that persisted up to FU6 (Pre: 12.3 ± 1.1 vs. FU1: 7.1 ± 1.1 and FU6: 6.3 ± 1.1; both *ps* < 0.001; see Fig. 5D).

## Discussion

The present proof-of principle feasibility study shows the efficacy of a remote-monitored, home-based AVT for persons with chronic HVFDs (average time since brain lesion ~ 29 months, range = 6–118 months). All participants complied with and adhered to the AVT without any interruption of the treatment; no adverse events or technical issues were reported.

In particular, the home-based AVT improves oculomotor visual scanning behavior (performance in the visual search tests, i.e., primary outcome) and reading, with positive outcomes on daily activities (v-ADLs) and mood. The effects of the training on reading and mood emerged over the long term. This finding suggests that the AVT benefits in daily life consolidate gradually after the end of treatment and then can persist up to 6 months post-training. Bolognini et al. [14] also demonstrated that the daily use of oculomotor compensation strategies implemented during treatment results in further gradual improvements in the ADLs on the long run, even months after the treatment completion.

Regarding mood, our study is the first to demonstrate a positive impact of the AVT on mood, although it should be noted that the sample did not exhibit a clinically significant depressive state prior to the training (mean baseline HDRS = 7.1). In contrast, a recent study [50] that investigated the long-term effects of a compensatory training on depression, as measured by the Geriatric Depression Scale [51], did not observe a treatment benefit on mood. However, even in that case, the presence of depressive symptoms in the sample was very low.

Of note, post-AVT improvements emerge net of the change observed during the training itself (namely, we did not detect a significant increase in audio-visual detection from Day 1 to Day 15 of the AVT). All participants were engaged in active training of saccadic eye movements towards various locations in the visual field. This daily exercise on a multisensory basis seems necessary to foster generalization to untrained visual scanning functions, as shown also in previous research [14, 15]. Overall, these findings confirm previous evidence on the efficacy of eye movement therapies based on multisensory stimulation [14, 15, 18, 19, 22], now documenting its clinical validity even when delivered at home under remote supervision. On the other hand, no enlargement of the visual field size or changes in VEPs (P100 latency and amplitude) or neuroimaging measurement were induced by the AVT, confirming its compensatory nature. However, only the most central 30 degrees of visual field were tested for each eye, so changes in the periphery cannot be excluded.

Interestingly, the baseline visual search performance (i.e., EF and Triangle tests) and the v-ADL score are both associated with post-treatment improvements: the more severe the visual exploration deficit and disability in daily life, the greater the training-induced benefits on both of these parameters. This finding is of key relevance because it suggests that greater benefits can be achieved in individuals presenting with severe deficits in visual scanning behavior, which preclude the development of successful oculomotor compensatory strategies.

The association between the severity of visual impairment and post-treatment improvements is further supported by the correlations between neurophysiological and neuroimaging data and post-treatment scanning abilities. Indeed, the participants with longer P100 latencies, smaller P100 amplitudes, or reduced optic radiation connectivity at baseline—which reflect more severe disturbances of low-level visual processing—exhibit greater post-AVT visual search improvements (EF and Number tests).

The efficacy of the AVT also relies on individual multisensory abilities: the more efficient the multisensory integration, as reflected by a narrower temporal binding window for audio-visual interactions [25, 52, 53], the greater the improvement in visual search brought about by the AVT. This last new finding is of main interest because it indicates the importance of assessing multisensory integration for choosing the optimal rehabilitation approach: if audio-visual stimuli cannot be efficiently integrated, their use for facilitating visual search and detection may be meaningless [23, 54]. In this regard, Frassinetti et al. [55] demonstrated that patients with HVFD show substantially spared multisensory integration abilities that aids visual perception in the blind hemifield. But if hemianopia is associated with visuo-spatial hemineglect [56], or if the lesion extends from occipital to parietal areas, multisensory stimuli have no facilitatory effect on visual perception.

The results of our second study further document that the effects produced by telerehabilitation with AVT are comparable to those achievable with the outpatient version of the AVT. It is worth noting that the AVT apparatus of the in-presence version subtended a larger portion of the visual field on the vertical axis and trained oculomotor responses to a larger number of spatial locations. This latter aspect probably explains the differences found between the two groups in terms of audio-visual detections during the training: participants in the in-person AVT had greater difficulty in detecting audio-visual stimuli in the blind hemifield, as compared to the accuracy rate of the participants who underwent the AVT at home. This also explains why, during the training, the improvement was detected in the in-person group (IP) but not in the home-trained group (HP see significant Group by Timepoint interaction, along with the significant main factor Group, indicating that audio-visual detections overall increase in both groups). We cannot exclude that if the HB group had been assessed and trained with same audio-visual panel as that of the IP group, we would have observed comparable results. Nonetheless, at baseline, the two groups did not differ in any visual search measure or v-ADL score, indicating a similar impact of vision loss on visual scanning and daily life.

Another difference between the HB and the IP treatments concerns the duration, two weeks for the outpatient AVT, three weeks for the in-person version. This difference was determined by clinical necessity: outpatient treatment duration is dictated by hospital organizational requirements. Future studies are needed to verify whether outpatient and at-home treatments, which are similar in intensity and structure, have the same effectiveness. However, we believe that telerehabilitation has the great advantage of allowing more intensive treatments at home, which might in any case be preferred to shorter outpatient treatments.

### On the advantages of telerehabilitation for HVFDs

In-person visual trainings are usually demanding and require travelling to the clinic several days a week, with a daily commitment of various hours, which may prevent acceptance of the treatment or adherence to it. This poses logistical problems for people in the chronic phase of the disease presenting with persistent deficits of visual exploration, who could benefit from additional treatment sessions to optimize their compensation strategies. Indeed, in-person therapies are usually incompatible with the daily routine that people with chronic HVFDs try to resume after discharge from the rehabilitation unit. Furthermore, living in rural and remote areas, or having severely reduced mobility with difficulty or inability to travel, poses additional logistical difficulties in reaching specialized rehabilitation centers, which are not particularly common when it comes to HVFDs. Telerehabilitation allows overcoming these and other limits, allowing the provision of clinical services to patients at remote locations via information and communication technologies [57]. The advantages of telerehabilitation are widely recognized for neurological disorders [31], and clinical trials testing its efficacy for post-stroke motor and cognitive impairments are increasing [29], in contrast with the paucity of attempts to verify its potential in the field of visual rehabilitation. The present study represents the first effort in this direction.

Our home-based AVT has the advantage of being easily transportable: the person with HVFD takes the AvDesk device home, which is rolled up and placed in a handy padded case that also contains the laptop computer that delivers the stimuli, records the data and sends it to the online server. Learning how to use the AvDesk requires a single short (approximately 30 min) training session. Importantly, the system allows constant remote supervision to monitor individual progress (hence possibly adjusting the therapy) and adherence to treatment, as well as to provide remote assistance for sudden technical problems. In terms of feasibility, in the present study, all participants managed to complete the training; only some of them, the older ones less familiar with the technology, needed the help of a caregiver to mount the setup at home. Overall, all the recruited participants demonstrated a good compliance: all of them followed the recommended training instructions and none of them dropped out during the treatment. The home-based AVT allows exercising in an intensive and active way, in a familiar context and with the chance to choose when performing the training during the day. This gives the person a sense of control and autonomy which contributes to compliance. On the other hand, the constant remote supervision also favors the feeling of being supported and not left alone. All these factors likely contribute to enhancing clinical outcomes, positively impacting mood, as shown here. In future studies, it will be important to evaluate how functional improvements in daily life translate into actual improvements in subjectively perceived quality of life, which is often reduced by vision loss following brain injury [7].

## Conclusion

The present study demonstrates the feasibility and the clinical efficacy of a novel telerehabilitation approach for chronic HVFDs. A 3-week, home-based, remotely-supervised AVT promotes the development of more efficient visual search to compensate for visual field loss, with long-lasting improvements and a positive impact on mood and vision-related disabilities in everyday life. The study also shows that the severity of visual scanning deficits and subsequent disability in activities of daily living, as well as the efficiency of multisensory integration, predict the treatment effects. These results shed light, for the first time, on predictors of the effectiveness of AVT, considering that these factors may be helpful in identifying the best candidates for this type of visual therapy.

## Supplementary Information

Additional file 1.

## Data Availability

The datasets generated and analyzed during the current study are available on Zenodo: https://doi.org/10.5281/zenodo.13335206. Datasets are accessible under request because they include sensitive information. Please write your request to the corresponding author.

## Abbreviations

AAL: Automated Anatomical Labelling atlas
ANOVA: Analysis of Variance
AVT: Audio-visual training
DTI: Diffusion tensor imaging
CSD: Constrained spherical deconvolution
CST: Cortico-spinal tract
FA: Fractional anisotropy
FU1: Follow-up assessment 1 month after the end of the training
FU6: Follow-up assessment 6 months after the end of the training
GMMs: Generalized mixed models
HDRS: Hamilton Depression Rating Scale
HH: Homonymous hemianopia
HVFD: Homonymous visual field defect
IFOF: Inferior fronto-occipital fasciculus
ILF: Inferior longitudinal fasciculus
IM: Group of participants who received immediate training
Inf: Inferior
LED: Light emitting diode
LMM: Linear Mixed Model
MD: Mean deviation in decibels at 30–2 visual field testing
MDiff: Mean diffusivity
MRI: Magnetic resonance imaging
OR: Optic radiations
OT: Optic tract
Post: Assessment immediately after the training
Pre: Baseline, i.e., pre-training assessment
Quad: Quadrantanopia
RTs: Response times
SLF: Superior longitudinal fasciculus
SJ2: Simultaneity audio-visual Judgement task
SPM: Statistical parametric mapping
TBW: Temporal binding window
v-ADLS: Vision-dependent activities of daily living
VEPs: Visual evoked potentials
WL: Wait-list group

## References

[1] Goodwin D. Homonymous hemianopia: challenges and solutions. Clin Ophthalmol. 2014;22(8):1919–27. 10.2147/OPTH.S59452 PMC4181645 25284978

[2] Ali M, Hazelton C, Lyden P, Pollock A, Brady M, VISTA Collaboration. Recovery from poststroke visual impairment: evidence from a clinical trials resource. Neurorehabil Neural Repair. 2013;27(2):133–41. 22961263 10.1177/1545968312454683

[3] Dogra N, Redmond BV, Lilley S, Johnson BA, Lam BL, Tamhankar M, et al. Vision-related quality of life after unilateral occipital stroke. Brain Behav. 2024;14(7): e3582. 38956813 10.1002/brb3.3582 PMC11219293

[4] Zihl J. Rehabilitation of visual disorders after brain injury. 1st ed. London: Psychology Press; 2021.

[5] de Haan GA, Heutink J, Melis-Dankers BJM, Brouwer WH, Tucha O. Difficulties in daily life reported by patients with homonymous visual field defects. J Neuroophthalmol. 2015;35(3):259–64. 25815856 10.1097/WNO.0000000000000244

[6] Ruddy J, Asuncion RMD, et al. Hemianopsia. In: Ruddy J, editor., et al., StatPearls. Treasure Island: StatPearls Publishing; 2024.

[7] Gall C, Lucklum J, Sabel BA, Franke GH. Vision- and health-related quality of life in patients with visual field loss after postchiasmatic lesions. Invest Ophthalmol Vis Sci. 2009;50(6):2765–76. 19117930 10.1167/iovs.08-2519

[8] Kempen GIJM, Ballemans J, Ranchor AV, van Rens GHMB, Zijlstra GAR. The impact of low vision on activities of daily living, symptoms of depression, feelings of anxiety and social support in community-living older adults seeking vision rehabilitation services. Qual Life Res. 2012;21(8):1405–11. 22090173 10.1007/s11136-011-0061-y PMC3438403

[9] Sabel BA, Wang J, Cardenas-Morales L, Faiq M, Heim C. Mental stress as consequence and cause of vision loss: the dawn of psychosomatic ophthalmology for preventive and personalized medicine. EPMA J. 2018;9(2):133–60. 29896314 10.1007/s13167-018-0136-8 PMC5972137

[10] Sabel BA, Thut G, Haueisen J, Henrich-Noack P, Herrmann CS, Hunold A, et al. Vision modulation, plasticity and restoration using non-invasive brain stimulation—an IFCN-sponsored review. Clin Neurophysiol. 2020;131(4):887–911. 32078919 10.1016/j.clinph.2020.01.008

[11] Pollock A, Hazelton C, Rowe FJ, Jonuscheit S, Kernohan A, Angilley J, et al. Interventions for visual field defects in people with stroke. Cochrane Database Syst Rev. 2019;5(5):8388. 10.1002/14651858.CD008388.pub3 PMC6532331 31120142

[12] Liu KPY, Hanly J, Fahey P, Fong SSM, Bye R. A systematic review and meta-analysis of rehabilitative interventions for unilateral spatial neglect and hemianopia poststroke from 2006 through 2016. Arch Phys Med Rehabil. 2019;100(5):956–79. 31030733 10.1016/j.apmr.2018.05.037

[13] Sahraie A, Cederblad AMH, Kenkel S, Romano JG. Efficacy and predictors of recovery of function after eye movement training in 296 hemianopic patients. Cortex. 2020;01(125):149–60. 10.1016/j.cortex.2019.12.005 31982700

[14] Bolognini N, Rasi F, Coccia M, Ladavas E. Visual search improvement in hemianopic patients after audio-visual stimulation. Brain. 2005;128(Pt 12):2830–42. 16219672 10.1093/brain/awh656

[15] Passamonti C, Bertini C, Ladavas E. Audio-visual stimulation improves oculomotor patterns in patients with hemianopia. Neuropsychologia. 2009;47(2):546–55. 18983860 10.1016/j.neuropsychologia.2008.10.008

[16] Keller I, Lefin-Rank G. Improvement of visual search after audiovisual exploration training in hemianopic patients. Neurorehabil Neural Repair. 2010;24(7):666–73. 20810740 10.1177/1545968310372774

[17] Dundon NM, Ladavas E, Maier ME, Bertini C. Multisensory stimulation in hemianopic patients boosts orienting responses to the hemianopic field and reduces attentional resources to the intact field. Restor Neurol Neurosci. 2015;33(4):405–19. 26409401 10.3233/RNN-140457

[18] Tinelli F, Purpura G, Cioni G. Audio-visual stimulation improves visual search abilities in hemianopia due to childhood acquired brain lesions. Multisens Res. 2015;28(1–2):153–71. 26152056 10.1163/22134808-00002484

[19] Tinelli F, Cioni G, Purpura G. Development and implementation of a new telerehabilitation system for audiovisual stimulation training in hemianopia. Front Neurol. 2017;21(8):621. 10.3389/fneur.2017.00621 PMC5702450 29209271

[20] Grasso PA, Ladavas E, Bertini C. Compensatory recovery after multisensory stimulation in hemianopic patients: behavioral and neurophysiological components. Front Syst Neurosci. 2016;24(10):45. 10.3389/fnsys.2016.00045 PMC4877493 27252629

[21] Diana L, Casati C, Melzi L, Marzoli SB, Bolognini N. Enhancing multisensory rehabilitation of visual field defects with transcranial direct current stimulation: a randomized clinical trial. Eur J Neurol. 2025;32(1): e16559. 39607286 10.1111/ene.16559 PMC11625917

[22] Alwashmi K, Meyer G, Rowe FJ. Audio-visual stimulation for visual compensatory functions in stroke survivors with visual field defect: a systematic review. Neurol Sci. 2022;43(4):2299–321. 35149925 10.1007/s10072-022-05926-y PMC8918177

[23] Bolognini N, Convento S, Rossetti A, Merabet LB. Multisensory processing after a brain damage: clues on post-injury crossmodal plasticity from neuropsychology. Neurosci Biobehav Rev. 2013;37(3):269–78. 23253947 10.1016/j.neubiorev.2012.12.006

[24] Bolognini N, Convento S, Casati C, Mancini F, Brighina F, Vallar G. Multisensory integration in hemianopia and unilateral spatial neglect: Evidence from the sound induced flash illusion. Neuropsychologia. 2016;01(87):134–43. 10.1016/j.neuropsychologia.2016.05.015 27197073

[25] Nava E, Giraud M, Bolognini N. The emergence of the multisensory brain: From the womb to the first steps. iScience. 2023;27(1):108758. 38230260 10.1016/j.isci.2023.108758 PMC10790096

[26] Bolognini N, Vallar G. Neglect, hemianopia and their rehabilitation. In: Sathian K, Ramachandran VS, editors. Multisensory perception: from laboratory to clinic. Amsterdam: Elsevier; 2019.

[27] Stein BE, Rowland BA. Using superior colliculus principles of multisensory integration to reverse hemianopia. Neuropsychologia. 2020;01(141): 107413. 10.1016/j.neuropsychologia.2020.107413 PMC9680976 32113921

[28] Bean NL, Stein BE, Rowland BA. Cross-modal exposure restores multisensory enhancement after hemianopia. Cereb Cortex. 2023;33(22):11036–46. 37724427 10.1093/cercor/bhad343 PMC10646694

[29] Laver KE, Adey-Wakeling Z, Crotty M, Lannin NA, George S, Sherrington C. Telerehabilitation services for stroke. Cochrane Database Syst Rev. 2020;1(1):CD010255. 32002991 10.1002/14651858.CD010255.pub3 PMC6992923

[30] Johansson T, Wild C. Telerehabilitation in stroke care—a systematic review. J Telemed Telecare. 2011;17(1):1–6. 21097560 10.1258/jtt.2010.100105

[31] Olowoyo P, Dhamija RK, Owolabi MO. Telerehabilitation—historical perspectives and conceptual framework in reference to neurological disorders: a narrative review. NeuroRehabilitation. 2024. 10.3233/NRE-240079. 38995808 10.3233/NRE-240079 PMC11902888

[32] Daibert-Nido M, Pyatova Y, Cheung K, Nayomi C, Markowitz SN, Bouffet E, et al. Case report: visual rehabilitation in hemianopia patients. Home-based visual rehabilitation in patients with hemianopia consecutive to brain tumor treatment: feasibility and potential effectiveness. Front Neurol. 2021;12:680211. 34354660 10.3389/fneur.2021.680211 PMC8333276

[33] Aimola L, Lane AR, Smith DT, Kerkhoff G, Ford GA, Schenk T. Efficacy and feasibility of home-based training for individuals with homonymous visual field defects. Neurorehabil Neural Repair. 2014;28(3):207–18. 24048623 10.1177/1545968313503219

[34] Cornoldi C, Candela M. Batteria MT 16–19. Batteria per la valutazione degli apprendimenti di lettura e scrittura e la diagnosi di dislessia e disortografia in ragazzi dai 16 ai 19 anni. Trento: Erickson; 2015.

[35] Kerkhoff G, Munssinger U, Meier EK. Neurovisual rehabilitation in cerebral blindness. Arch Neurol. 1994;51(5):474–81. 8179497 10.1001/archneur.1994.00540170050016

[36] Hamilton M. A rating scale for depression. J Neurol Neurosurg Psychiatry. 1960;23(1):56–62. 14399272 10.1136/jnnp.23.1.56 PMC495331

[37] van Eijk RLJ, Kohlrausch A, Juola JF, van de Par S. Audiovisual synchrony and temporal order judgments: effects of experimental method and stimulus type. Percept Psychophys. 2008;70(6):955–68. 18717383 10.3758/pp.70.6.955

[38] Giurgola S, Casati C, Stampatori C, Perucca L, Mattioli F, Vallar G, et al. Abnormal multisensory integration in relapsing-remitting multiple sclerosis. Exp Brain Res. 2022;240(3):953–68. 35094114 10.1007/s00221-022-06310-0 PMC8918188

[39] Stevenson RA, Segers M, Ferber S, Barense MD, Camarata S, Wallace MT. Keeping time in the brain: autism spectrum disorder and audiovisual temporal processing. Autism Res. 2016;9(7):720–38. 26402725 10.1002/aur.1566

[40] Rorden C, Brett M. Stereotaxic display of brain lesions. Behav Neurol. 2000;12(4):191–200. 11568431 10.1155/2000/421719

[41] Rorden C, Bonilha L, Fridriksson J, Bender B, Karnath H. Age-specific CT and MRI templates for spatial normalization. Neuroimage. 2012;61(4):957–65. 22440645 10.1016/j.neuroimage.2012.03.020 PMC3376197

[42] Penny WD, Friston KJ, Ashburner JT, Kiebel SJ, Nichols TE. Statistical parametric mapping: the analysis of functional brain images. Amsterdam: Elsevier; 2011.

[43] Tzourio-Mazoyer N, Landeau B, Papathanassiou D, Crivello F, Etard O, Delcroix N, et al. Automated anatomical labeling of activations in SPM using a macroscopic anatomical parcellation of the MNI MRI single-subject brain. Neuroimage. 2002;15(1):273–89. 11771995 10.1006/nimg.2001.0978

[44] Diana L, Casati C, Melzi L, Bianchi Marzoli S, Bolognini N. The effects of occipital and parietal tDCS on chronic visual field defects after brain injury. Front Neurol. 2024;14(15):1340365. 10.3389/fneur.2024.1340365 PMC10899507 38419713

[45] Tournier J, Calamante F, Connelly A. Robust determination of the fibre orientation distribution in diffusion MRI: non-negativity constrained super-resolved spherical deconvolution. Neuroimage. 2007;35(4):1459–72. 17379540 10.1016/j.neuroimage.2007.02.016

[46] Jeurissen B, Tournier J, Dhollander T, Connelly A, Sijbers J. Multi-tissue constrained spherical deconvolution for improved analysis of multi-shell diffusion MRI data. Neuroimage. 2014;01(103):411–26. 10.1016/j.neuroimage.2014.07.061 25109526

[47] Dhollander T, Connelly A. Generating a T1-like contrast using 3-tissue constrained spherical deconvolution results from single-shell (or multi-shell) diffusion MR data. ISMRM workshop on breaking the barriers of diffusion MRI. lisbon: Intl. soc. mag. reson. med. 2016.

[48] The jamovi project. jamovi. 2024; 2.5.

[49] Diana L, Pilastro P, Aiello NE, Eberhard-Moscicka AK, Müri RM, Bolognini N. Saccades, attentional orienting and disengagement: the effects of anodal tDCS over right posterior parietal cortex (PPC) and frontal eye field (FEF). ACM symposium on eye tracking research and applications. 2021.

[50] Zihl J, Kentridge RW, Pargent F, Heywood CA. Aging and the rehabilitation of homonymous hemianopia: the efficacy of compensatory eye-movement training techniques and a five-year follow up. Aging Brain. 2021;23(1): 100012. 10.1016/j.nbas.2021.100012 PMC9997164 36911515

[51] Yesavage JA, Brink TL, Rose TL, Lum O, Huang V, Adey M, et al. Development and validation of a geriatric depression screening scale: a preliminary report. J Psychiatr Res. 1982;17(1):37–49. 7183759 10.1016/0022-3956(82)90033-4

[52] Zhou H, Cheung EFC, Chan RCK. Audiovisual temporal integration: cognitive processing, neural mechanisms, developmental trajectory and potential interventions. Neuropsychologia. 2020;16(140): 107396. 10.1016/j.neuropsychologia.2020.107396 32087206

[53] Sanders P, Thompson B, Corballis P, Searchfield G. On the timing of signals in multisensory integration and crossmodal interactions: a scoping review. Multisens Res. 2019;32(6):533–73. 31137004 10.1163/22134808-20191331

[54] Bolognini N, Russo C, Vallar G. Crossmodal illusions in neurorehabilitation. Front Behav Neurosci. 2015;10(9):212. 10.3389/fnbeh.2015.00212 PMC4530305 26321933

[55] Frassinetti F, Bolognini N, Bottari D, Bonora A, Ladavas E. Audiovisual integration in patients with visual deficit. J Cogn Neurosci. 2005;17(9):1442–52. 16197697 10.1162/0898929054985446

[56] Vallar G, Bolognini N. Unilateral Spatial Neglect. In: Nobre AC, Kastner S, editors. Oxford Handbook of Attention. Oxford: Oxford University Press; 2014.

[57] Brennan DM, Mawson S, Brownsell S. Telerehabilitation: enabling the remote delivery of healthcare, rehabilitation, and self management. Stud Health Technol Inform. 2009;145:231–48. 19592797

