# Supplementary material for: Telerehabilitation for visual field defects with a multisensory training: a feasibility study
Source: J Neuroeng Rehabil. 2025 Feb 24;22:34. doi: 10.1186/s12984-025-01573-4 (PMC11849177; doi:10.1186/s12984-025-01573-4)
Supplement: Supplementary file 1 — Additional file 1. [file 12984_2025_1573_MOESM1_ESM.docx]

Supplementary Materials

**STUDY 1: CLINICAL EFFICACY OF TELEREHABILITATION FOR VISUAL FIELD DEFECTS**

Table S1 reports detailed clinical-demographic characteristics of the group who began the treatment immediately (IM) and the wait-list group (WL) at baseline (Pre assessment).

Table S1. *Detailed* *clinical-demographic characteristics of the immediate treatment group (IM) and the wait-list group (WL) at baseline (Pre assessment).*

|  | **IM** | **WL** |
| --- | --- | --- |
| **Age (years)** | 54.2 ± 14.5 | 54.2 ± 18.6 |
| **Sex** | 8 females, 5 males | 6 females, 7 males |
| **Disease duration (month)** | 24.1 ± 16.8 | 33.2 ± 31.2 |
| **Etiology** | 3 hemorrhagic stroke  8 ischemic stroke  2 TBI | 7 hemorrhagic stroke  4 ischemic stroke  1 TBI  1 brain tumor |
| **Visual field size (MD, db)** | -11.2 ± 5.3 | -14 ± 2.7 |
| **Visual field defect** | 1 left inf. quad.  8 left HH  4 right HH | 1 left inf. quad.  5 left HH  7 right HH |
| **Lesion volume (cm^3^)** | 23.1 ± 29.1 | 35.6 ± 37.6 |
| **EF test – accuracy (%)** | 83.8 ± 21.4 | 88.3 ± 12.2 % |
| **Triangles Test – accuracy (%)** | 79.6 ± 13.2 | 77.2 ± 15.4 % |
| **Numbers Test – RTs (ms)** | 34657 ± 8156 | 34078 ± 12280 |
| **Reading speed (syllables/sec.)** | 3.9 ± 1.8 | 3.6 ± 1.5 |
| **v-ADLs** | 11.5 ± 7.8 | 9.2 ± 4.6 |
| **HDRS** | 7.2 ± 4.2 | 7 ± 6 |
| *Note: N*europsychological and neuro-ophthalmological results refer to the baseline assessment, i.e., timepoint Pre. For quantitative variables, mean ± standard deviation is reported.  MD= mean deviation in decibels (db) at 30-2 visual field testing; inf= inferior; quad= quadrantanopia; TBI= traumatic brain injury; HH= homonymous hemianopia; RTs= response times; v-ADLs= vision-dependent activities of daily living; HDRS= Hamilton Depression Rating Scale. | | |

**Post-treatment changes in visual search speed for the EF and the Triangles test**

A Generalized Mixed Models (GLM) with fixed-factor Timepoint (Pre, Post, FU1, and FU6) and Test (EF and Triangles) showed an effect of Timepoint (*X²* = 53.7, *p* < 0.001), Test (*X²* = 723, *p* < 0.001), and a Timepoint X Test interaction (*X²* = 24.2, *p* < 0.001).

Of relevance, Holm-corrected post-hocs for Timepoint showed small but significant improvements in visual search speed emerging at FU1 (Mean ± SE = 5876 ± 93.6 ms; *p* = 0.006) and at FU6 (5760 ± 80.8 ms; *p* < 0.001), compared to Pre (6135 ± 65.8 ms). See also Table S2. The significant effect of Test showed that participants were overall faster in the EF test (4738 ± 77.6 ms) compared to the Triangles test (7106 ± 75.4 ms). Lastly, the Timepoint x Test interaction showed that, for the EF test, a significant improvement was observed only at FU6 as compared to the baseline performance (*p* = 0.003), whereas for the Triangles test a significant post-treatment speed-up of RTs was observed at FU1 (*p* < 0.001) and was maintained at the 6-month follow-up (*p* < 0.001).

| Table S2. Post Hoc Comparisons - Timepoint | | | | | | | | | | | | | |
| --- | --- | --- | --- | --- | --- | --- | --- | --- | --- | --- | --- | --- | --- |
| **Comparison** | | | | | |  | | | | | | | |
| **Timepoint** | |  | | **Timepoint** | | **Difference** | | **SE** | | **z** | | **p_holm_** | |
| Pre |  | - |  | Post |  | 218.2 |  | 97.5 |  | 2.238 |  | 0.101 |  |
| Pre |  | - |  | FU1 |  | 259.4 |  | 79.8 |  | 3.249 |  | 0.006 |  |
| Pre |  | - |  | FU6 |  | 375.1 |  | 59.7 |  | 6.279 |  | < .001 |  |
| Post |  | - |  | FU1 |  | 41.2 |  | 140.2 |  | 0.294 |  | 0.769 |  |
| Post |  | - |  | FU6 |  | 156.9 |  | 118.6 |  | 1.323 |  | 0.557 |  |
| FU1 |  | - |  | FU6 |  | 115.8 |  | 87.9 |  | 1.317 |  | 0.557 |  |
|  | | | | | | | | | | | | | |

**Predictors of treatment efficacy**

Table S3 reports all Spearman correlations between clinical and experimental baseline performance and post-treatment changes in neuropsychological outcomes.

Table S3. *Predictors of treatment efficacy – table of correlations*

|  | **ΔPost-Pre**  **EF Acc** | **ΔPost-Pre**  **Tri Acc** | **ΔPost-Pre**  **Numb RTs** | **ΔPost-Pre**  **v-ADLs** |
| --- | --- | --- | --- | --- |
| **Baseline performance^a^** | ***rs* = -0.759**  ***p* < .001** | ***rs* = -0.622**  ***p* < .001** | *rs* = -0.303  *p* = 0.133 | ***rs* = -0.539**  ***p* = 0.005** |
| **Age (years)** | *rs* = 0.311  *p* = 0.122 | *rs* = 0.172  *p* = 0.402 | *rs* = -0.178  *p* = 0.385 | *rs* = 0.187  *p* = 0.36 |
| **Disease duration (months)** | *rs* = -0.228  *p* = 0.263 | *rs* = 0.064  *p* = 0.756 | *rs* = 0.053  *p* = 0.798 | *rs* = -0.094  *p* = 0.647 |
| **Visual field size (MD, db)^b^** | *rs* = -0.03  *p* = 0.883 | *rs* = -0.177  *p* = 0.387 | *rs* = 0.024  *p* = 0.91 | *rs* = -0.237  *p* = 0.243 |
| **P100 amplitude (µV), blind hemifield** | ***rs* = -0.447**  ***p* = 0.022** | *rs* = -0.222  *p* = 0.275 | *rs* = -0.017  *p* = 0.933 | *rs* = -0.181  *p* = 0.375 |
| **P100 latency (ms), blind hemifield** | *rs* = 0.097  *p* = 0.636 | ***rs* = 0.496**  ***p* = 0.01** | *rs* = 0.001  *p* = 0.999 | *rs* = 0.032  *p* = 0.876 |
| **TBW** | ***rs* = -0.492**  ***p* = 0.029** | *rs* = 0.112  *p* = 0.638 | *rs* = 0.083  *p* = 0.729 | *rs* = 0.067  *p* = 0.779 |
| **Lesion Volume (cm^3^)** | *rs* = 0.082  *p* = 0.724 | *rs* = 0.19  *p* = 0.408 | *rs* = 0.174  *p* = 0.449 | *rs* = 0.142  *p* = 0.541 |
| **Occipital lesion extension (voxels)** | *rs* = 0.354  *p* = 0.116 | *rs* = 0.082  *p* = 0.725 | *rs* = 0.051  *p* = 0.826 | *rs* = 0.158  *p* = 0.493 |
| **Temporal lesion extension (voxels)** | *rs* = 0.258  *p* = 0.258 | *rs* = 0.029  *p* = 0.9 | *rs* = 0.001  *p* = 0.999 | *rs* = 0.029  *p* = 0.902 |
| **Parietal lesion extension (voxels)** | *rs* = 0.048  *p* = 0.835 | *rs* = 0.098  *p* = 0.672 | *rs* = 0.015  *p* = 0.947 | *rs* = -0.256  *p* = 0.263 |
| **ILF FA** | *rs* = -0.133  *p* = 0.566 | *rs* = -0.084  *p* = 0.718 | *rs* = -0.131  *p* = 0.572 | *rs* = -0.229  *p* = 0.319 |
| **ILF MDiff** | *rs* = -0.127  *p* = 0.584 | *rs* = 0.268  *p* = 0.241 | *rs* = 0.294  *p* = 0.196 | *rs* = 0.164  *p* = 0.477 |
| **SLF FA** | *rs* = -0.172  *p* = 0.455 | *rs* = 0.086  *p* = 0.712 | *rs* = -0.215  *p* = 0.35 | *rs* = -0.297  *p* = 0.191 |
| **SLF MDiff** | *rs* = -0.081  *p* = 0.726 | *rs* = 0.15  *p* = 0.518 | *rs* = 0.212  *p* = 0.357 | *rs* = 0.17  *p* = 0.461 |
| **OR FA** | *rs* = -0.006  *p* = 0.98 | *rs* = -0.083  *p* = 0.728 | *rs* = -0.034  *p* = 0.886 | *rs* = -0.199  *p* = 0.399 |
| **OR MDiff** | *rs* = -0.383  *p* = 0.096 | *rs* = 0.113  *p* = 0.635 | ***rs* = 0.574**  ***p* = 0.008** | *rs* = 0.193  *p* = 0.414 |
| **OT FA** | *rs* = -0.004  *p* = 0.989 | *rs* = -0.312  *p* = 0.223 | *rs* = 0.317  *p* = 0.216 | *rs* = -0.466  *p* = 0.06 |
| **OT MDiff** | *rs* = 0.015  *p* = 0.959 | *rs* = 0.326  *p* = 0.201 | *rs* = -0.353  *p* = 0.165 | *rs* = 0.081  *p* = 0.757 |
| **IFOF FA** | *rs* = -0.302  *p* = 0.183 | *rs* = -0.074  *p* = 0.75 | *rs* = 0.022  *p* = 0.925 | *rs* = -0.181  *p* = 0.432 |
| **IFOF MDiff** | *rs* = 0.02  *p* = 0.93 | *rs* = 0.313  *p* = 0.167 | *rs* = 0.207  *p* = 0.367 | *rs* = 0.116  *p* = 0.617 |
| *Legend*: significant correlations are reported in bold.  ^a^ Baseline value (pre-treatment values, Pre) of each test/scale is only correlated with the ΔPost-Pre change of the respective test/scale; ^b^ mean deviation (MD) in decibels (db) at 30-2 visual field testing, measured at Pre;  FA = factional anisotropy of the tract in the lesioned hemisphere, pre-treatment brain scan; ILF = inferior longitudinal fasciculus; MDiff = mean diffusivity of the tract in the lesioned hemisphere, pre-treatment brain scan; OR = optic radiations; OT = optic tract; SLF = superior longitudinal fasciculus; TBW = Amplitude of the temporal binding window (i.e., efficiency of the multisensory integration), measured at Pre. | | | | |

**STUDY 2: COMPARISON OF HOME-BASED AND IN-PERSON VERSIONS OF THE AVT**

**Post-treatment changes in visual search speed for the EF and the Triangles test**

A Linear Mixed Model with fixed-factor Timepoint (Pre, Post, FU1, and FU6), Test (EF and Triangles), and Group (HB vs IP) showed an effect of Test (*F_1,183.1_* = 408.4, *p* < 0.001), and a Test X Group interaction (*F_1,183.1_* = 24.1, *p* < 0.001). All other main effects and interactions did not reach the significance level (all *F* < 2.4; all *ps* > 0.07).

Holm-corrected post-hocs for the factor Test showed overall faster performance for the EF test (Mean ± SE = 4328 ± 358 ms) compared to the Triangles test (7209 ± 358 ms). The Test X Group interaction further confirmed the previous finding (i.e., both in the HB and the IP groups, the performance was faster in the EF test than in the Triangles test; both ps < 0.001), but no between-group differences were observed (HB vs. IP; EF test: *p* = 0.8; Triangles test: *p* = 0.2), demonstrating comparable performance in both groups.
